# Overexpression of the microtubule-binding protein CLIP-170 induces a +TIP network superstructure consistent with a biomolecular condensate

**DOI:** 10.1371/journal.pone.0260401

**Published:** 2021-12-10

**Authors:** Yueh-Fu O. Wu, Annamarie T. Bryant, Nora T. Nelson, Alexander G. Madey, Gail F. Fernandes, Holly V. Goodson

**Affiliations:** 1 Department of Chemistry and Biochemistry, University of Notre Dame, Notre Dame, IN, United States of America; 2 Integrated Biomedical Sciences Graduate Program, University of Notre Dame, Notre Dame, IN, United States of America; 3 Department of Biological Sciences, University of Notre Dame, Notre Dame, IN, United States of America; University of Virginia School of Medicine, UNITED STATES

## Abstract

Proper regulation of microtubule (MT) dynamics is critical for cellular processes including cell division and intracellular transport. Plus-end tracking proteins (+TIPs) dynamically track growing MTs and play a key role in MT regulation. +TIPs participate in a complex web of intra- and inter- molecular interactions known as the +TIP network. Hypotheses addressing the purpose of +TIP:+TIP interactions include relieving +TIP autoinhibition and localizing MT regulators to growing MT ends. In addition, we have proposed that the web of +TIP:+TIP interactions has a physical purpose: creating a dynamic scaffold that constrains the structural fluctuations of the fragile MT tip and thus acts as a polymerization chaperone. Here we examine the possibility that this proposed scaffold is a biomolecular condensate (i.e., liquid droplet). Many animal +TIP network proteins are multivalent and have intrinsically disordered regions, features commonly found in biomolecular condensates. Moreover, previous studies have shown that overexpression of the +TIP CLIP-170 induces large “patch” structures containing CLIP-170 and other +TIPs; we hypothesized that these structures might be biomolecular condensates. To test this hypothesis, we used video microscopy, immunofluorescence staining, and Fluorescence Recovery After Photobleaching (FRAP). Our data show that the CLIP-170-induced patches have hallmarks indicative of a biomolecular condensate, one that contains +TIP proteins and excludes other known condensate markers. Moreover, bioinformatic studies demonstrate that the presence of intrinsically disordered regions is conserved in key +TIPs, implying that these regions are functionally significant. Together, these results indicate that the CLIP-170 induced patches in cells are phase-separated liquid condensates and raise the possibility that the endogenous +TIP network might form a liquid droplet at MT ends or other +TIP locations.

## Introduction

Microtubules (MTs) compose one of the three major filament networks of the eukaryotic cytoskeleton, and they are required for basic cellular functions such as cell polarity, cell division, and intracellular transport. Dysfunction of the MT cytoskeleton can lead to serious neurodegenerative diseases including tauopathies and Parkinson’s disease, and compounds that target MTs are significant as chemotherapy agents, fungicides, and herbicides [1,2].

Microtubules display a surprising behavior known as dynamic instability, which describes the approximately random alteration between phases of slow growth (polymerization) and rapid shrinkage (depolymerization). This behavior is regulated by MT binding proteins and is central to MT function because it enables MTs to explore space to respond rapidly to internal and external signals and find organelles to be transported (reviewed by [2]).

**Fig 1:**
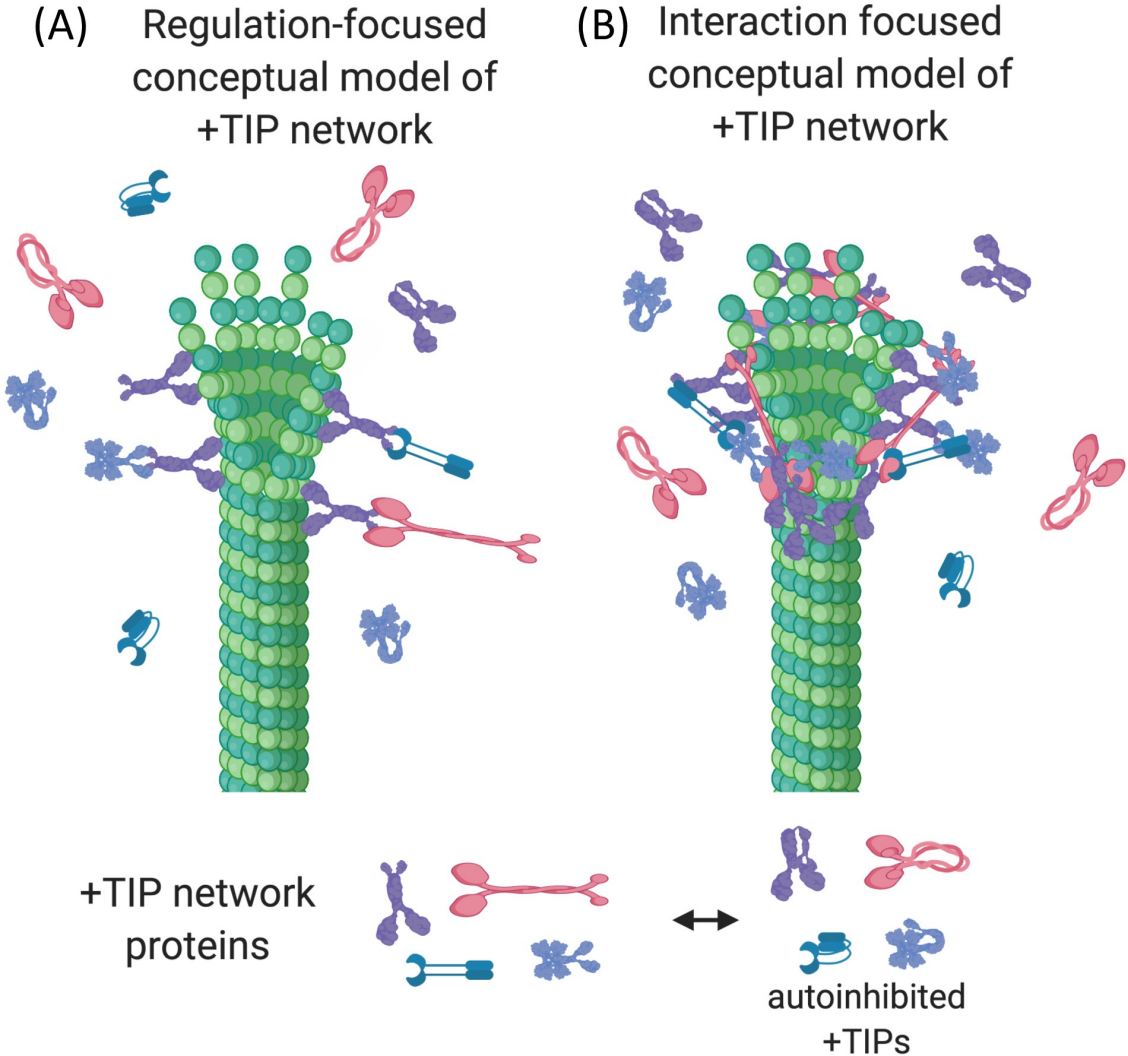
Conceptual models of the +TIP network. (A) In the regulation-focused model of the +TIP network, interactions between +TIPs function primarily to activate autoinhibited +TIP proteins and/or enable +TIPs to localize to the plus end. (B) In a more physical model of the +TIP network, interactions between +TIP proteins form a cross-linked, dynamic scaffold that supports and stabilizes the fluctuating MT tips by promoting lateral binding of protofilaments [4]. This figure was created with BioRender [5].

The most conserved MT binding proteins (and by implication the most important) are a set of mutually interacting proteins that dynamically track growing MT ends and are collectively known as microtubule plus-end tracking proteins (+TIPs). +TIPs form an interaction network created by many weak, multivalent links (both intra- and inter-molecular) between MTs and +TIPs [2,3]. While many +TIPs and their MT regulatory roles have been identified, it is not yet fully understood why so many +TIPs bind to other +TIPs. One favored explanation is that the interactions of the +TIP network create regulatory pathways by relieving the autoinhibition feature present in many +TIP proteins (Fig 1A). Another explanation is that +TIP:+TIP interactions serve to localize and deliver proteins in a spatiotemporal manner (e.g. localizing +TIPs to the MT ends, and facilitating the surfing of proteins to cell edge) (reviewed in [3]).

While these explanations for the existence of the +TIP network are logical, some aspects of +TIP behavior seem inconsistent with either idea, suggesting that this set of explanations is not complete. For example, deletion of evolutionarily conserved +TIPs often has surprisingly little effect on MT dynamics and/or cell survival (S1 Table in S5 File), which is especially surprising if the localization and/or activity of some conserved +TIPs depend on others. In addition, while individual +TIPs can function independently in regulating MT dynamics, evidence from several labs also suggests that groups of +TIPs work synergistically to promote MT polymerization [4,6,7]. Such synergy seems inconsistent with linear regulatory and/or localization pathways, which would predict epistasis or at most additive effects. These observations led us to suggest in earlier work that the interactions of the +TIP network have an important additional function, which is to create a superstructure that promotes MT assembly by constraining the structural fluctuations of the MT tip (Fig 1B) [4].

The work in this paper focuses on the +TIP CLIP-170, which was the first characterized +TIP [8]. Previous studies have found that CLIP-170 promotes interactions between organelles and MTs [8–10] and regulates MT dynamics [11]. CLIP-170 has also been implicated in actin-MT crosstalk (e.g., [12–14]). Similar to other +TIPs, CLIP-170 has multiple conserved binding sites that mediate interactions with MTs and/or other +TIPs [15,16]. Structurally, CLIP-170 contains three major domains. The N-terminal head domain is composed of two CAP-Gly subdomains and three serine-rich regions, all of which contribute to MT binding [15]. The coiled-coil region mediates CLIP-170 dimerization [17] and contains a FEED domain, which forms a complex with formin and actin to promote actin polymerization [12]. The C-terminal domain contains two zinc-knuckle motifs [9,17,18]: the first of these mediates CLIP-170:CLIP-170 interactions involved in autoinhibition, while the second mediates an interaction with the dynein-associated dynactin complex [19,20].

Overexpression of CLIP-170 causes formation of so-called “patches” [9,19]. The physical identity of these patches was puzzling to the researchers performing early work on CLIP-170, who initially assumed that the patches were membranous organelles of some kind but were not able to colocalize membrane markers to them. These early efforts showed that CLIP-170 could recruit other proteins more recently recognized as +TIPs (specifically EB1 and dynactin complex) into the patches [19]. These characteristics and others described below led us to speculate that the patches might correspond to biomolecular condensates. The goal of the work described in this paper is to investigate this possibility and consider its potential implications in terms of the form and function of the +TIP network under normal physiological conditions.

Biomolecular condensates (aka liquid droplets, coacervates, membraneless organelles) are dynamic structures that spontaneously form when interactions between groups of weakly-binding biopolymers (protein and sometimes RNA or DNA) cause them to phase-separate from surrounding cytoplasm or nucleoplasm [21–24]. Cytosolic examples include P-bodies, which regulate mRNA turnover [25,26], Wnt signalosomes, which help to transduce Wnt signaling [27–29], and the pericentriolar material/matrix, which regulates MT nucleation at the centrosome [30,31]. In the nucleus, Cajal bodies regulate RNA-related metabolism [32], and PML bodies regulate nuclear functions [33]. While these biomolecular condensates are associated with functions that promote cell survival, not all are positive: condensates of the MT-associated protein Tau corelate with the progression of Alzheimer’s disease [34–36].

The process in which proteins/nucleic acids self-assemble into a condensate is called liquid-liquid phase separation (LLPS). This liquid-liquid demixing process becomes observable when the proteins/nucleic acids reach a critical concentration [21–24]. Biomolecules identified as forming condensates generally contain intrinsically disordered regions (IDRs) and multivalent binding regions that mediate weak interactions. These sequence features generate the driving forces for condensate formation [21–24]. Physical factors such as temperature and pH can affect the condensation or dissolution of LLPS as well [21–24].

While many condensates are now well-recognized [21,24], there are relatively few examples of mechanistically defined functions for condensate formation. Proposed functions include concentrating reactive molecules locally to promote cellular reactions (e.g., promoting actin nucleation [37]), serving as a storage depot (e.g., P-granules [25]), and providing mechanical forces to generate local viscoelasticity, which can be important to resist the deformation of cellular structures (e.g. condensate formation at the centrosome [38]).

Even though the functions and localizations of these condensates are varied, some attributes are shared between them. Common hallmarks that are thought to identify a biomolecular condensate include a) elastic deformability, b) the ability to undergo fusion and fission, c) selectivity (i.e., inclusion of certain biomolecules and the exclusion of others), and d) rapid exchange of proteins between the droplets and the cytoplasm [21–23,39].

Here, we use live-cell imaging, photobleaching, and immunofluorescence to show that the “patches” previously observed as being induced upon CLIP-170 overexpression have the four hallmarks of liquid-liquid condensates, leading us to conclude that the patches are biomolecular condensates. We also evaluated the sequence properties of CLIP-170 and other +TIPs for the presence of sequence characteristics associated with condensate formation. We show that many +TIPs both are multivalent and contain IDRs, and that for key +TIPs these sequence features are conserved across large spans of evolution, suggesting that they are functionally significant. These data are consistent with our previous proposal that the +TIP network forms a physical superstructure and lead us to speculate that this superstructure is (in at least some circumstances or locations) a biomolecular condensate that coats the MT tip to support and promote MT assembly.

## Materials and methods

### Cell lines and cell culture

NIH3T3 cells (a gift of Dr. Reginal Hill) were grown in Dulbecco’s Modified Eagle Medium (DMEM, Sigma) plus 2% glutamine (BioWhittaker) and 10% bovine calf serum (VWR). Cos-7 cells (a gift of Dr. Kevin Vaughan) were grown in DMEM plus 1% glutamine and 10% fetal bovine serum (Sigma). Cells were incubated under standard cell culture conditions (37°C and 5% CO_2_).

### DNA constructs

CLIP-170 constructs utilize the version of CLIP-170 used in the original Kreis lab publication [8], now described in accession AAA35693. CLIP-170 plasmids with expressed transgenes under control of the CMV promoter have been described previously: WT CLIP-170 in pSG5 vector (pSG5-myc-CLIP-170) [40], and the N-terminal EGFP-conjugated WT CLIP-170 in pCB6 vector (pCB6-GFP-CLIP-170, “GFP-CLIP-170” here after) [41]. mEmerald-CLIP170-N-18, hereafter called N-18, was a gift from Michael Davidson (Addgene plasmid # 54044). N-18 has an mEmerald tag at the C-terminus. The N-18 GSSG construct lacks the mEmerald but has extra 4 amino acid GSSG tag at the C-terminus of CLIP-170, while the N-18 ETF construct has a stop codon after the native C-terminus (ETF) of CLIP-170.

### Antibodies & lipid dyes

The CLIP-170 antibodies used in this study were either a rabbit polyclonal raised against *Xenopus* CLIP-170 (this manuscript) or the mixture of mouse monoclonal antibodies 4D3/2D6 [8,42], with the exception of the GalT colocalization experiment, which utilized the rabbit polyclonal ɑ55 raised against human CLIP-170 [8]. p150 was recognized by rabbit polyclonal anti-p150^Glued^, which was a gift from K. Vaughan and R. Vallee. EEA1 was recognized by rabbit polyclonal anti-EEA1 [43]. The monoclonal antibody against GalT was a gift to Dr. Thomas Kris from Dr. T. Suganuma [44]. Other primary antibodies were obtained from commercial sources: hnRNPA1—mouse hnRNPA1 monoclonal antibody 4B10 (Invitrogen, MA5-24774), YB1—rabbit YB1 polyclonal antibody D299 (Cell Signaling, 4202S), Staufen1—rabbit polyclonal antibody (Bios antibodies, bs-9877R), CLASP1—rat monoclonal antibody (Absea, KT66), CLASP2—rat monoclonal antibody (Absea, KT69), mDia2—rabbit DIAPH3 polyclonal antibody (Proteintech, 14342-1-AP), EB1—mouse monoclonal antibody (Transduction Laboratories, now BD-Transduction, 610534), LIS-1—mouse monoclonal antibody H-7 (Santa Cruz, sc-374586), and tubulin–mouse monoclonal antibody 1A2 (Sigma, T9028). All labeled secondary antibodies were obtained from Invitrogen. Generic RNA was stained with SYTO™ RNASelect™ Green Fluorescent Cell Stain (Thermo Fisher, S32703). Rhodamine DHPE (Thermo Fisher, L-1392), Dil (Invitrogen), and RhPE (Molecular Probes) were used as generic membrane labels. LysoTracker (Molecular Probes, DND-99) and MitoTracker Red CMXRos (Cell Signaling) were used to stain lysosomes and mitochondria, respectively. For all membrane staining experiments, cells were stained according to manufacturer protocols, and then moved into imaging medium for imaging.

### Transient transfections

For fixed cell images, cells were grown on 10 mm^2^ glass coverslips (Knittel Glaser). For live cell imaging, cells were grown on 35 mm dishes with 10 mm glass bottom coverslips (P35G-1.5-10-C, MatTek) or 8-well glass-bottom chambers (Eppendorf). Cells were transiently transfected by Lipofectamine 3000 (Invitrogen, L3000008) with 5 μg of indicated construct DNA per reaction when cells reached 70–80% confluence. For consistency, cells were incubated for 24 hr post-transfection before performing live imaging, FRAP, and immunofluorescence assays unless otherwise indicated.

### Heat shock and drug treatment

Cells that underwent heat shock or drug treatments are indicated in their figure legends. To induce stress granules, NIH3T3 cells were incubated at 42°C or treated with 0.5 mM NaAsO_2_ for one hour before fixation as indicated in the figure legend. To study the relationship between CLIP-170-induced patches and tubulin/MTs, NIH3T3 cells were incubated with 5 μM Nocodazole for 16 hr or 1 hr before fixation as indicated in the figure legend. For consistency, all cells were incubated for 24 hr post-transfection before fixation unless otherwise indicated, regardless of possible heat shock or drug treatment.

### Immunofluorescence

As indicated in the figure legends, cells were fixed by methanol or 3% paraformaldehyde (PFA) before staining with the antibodies described above. For the methanol fixation, cells were fixed in -20°C methanol for 4 min and washed three times with PBS before treatment with primary antibodies. For the PFA fixation, cells were fixed in 3% PFA for 20 min, washed once with PBS, quenched with a wash of 30 mM ammonium chloride in PBS, and washed once again in PBS. Cells were then permeabilized with 0.1% Triton-X-100 for 4 min and washed with PBS again before treating with primary antibodies. Each washing step was 5 min. Cells were incubated with the primary antibody for 20 min, washed three times with PBS, incubated with the indicated fluorophore-conjugated secondary antibodies for 20 min, and washed three times with PBS. Cells were mounted on slides in Mowiol 4–88 mounting medium (Sigma 475904-M). Unless otherwise indicated, images were acquired with a 100x objective (1.4 N.A.) and a 1.5× optivar on a TE2000 inverted microscope (Nikon) with a Hamamatsu CMOS camera controlled by NIS-Elements BR 413.04 64-bit software (Nikon). Alternatively, microscopy was performed on a DeltaVision Deconvolution Microscope (GE Healthcare) with a 60x 1.42 N.A. objective connected to a Photometrics Coolsnap hq2 monochrome camera controlled by Resolve3D –SoftWoRx 7.0.0 software (GE Healthcare). Images were processed using Fiji (National Institutes of Health, https://imagej.net/Fiji) [45]. For the EEA1 and GalT immunofluorescence experiments in Supplementary Information, cells were imaged using a 40x 1.4NA PlanApo objective on a Zeiss inverted microscope (TV135); images were recorded with a cooled CCD camera (Photometrics CH250, Tuscon AZ) driven by IPLab-Spectrum software (Scanalytics, Fairfax VA), and processed by Photoshop (Adobe). Because CLIP-170 patches can be very bright, negative controls with one primary and two secondary antibodies were routinely performed to assess levels of signal created by bleed through and/or antibody cross-reactivity.

### Live imaging and Fluorescence Recovery After Photobleaching (FRAP)

Live imaging experiments were performed either with an A1R-MP Laser Scanning Confocal Microscope or with DeltaVision as indicated. In both systems, cells were incubated at 37°C with 0.5% CO_2_ within an environmental chamber. DMEM culture media lacking phenol red (Sigma, 51449C) plus 2% glutamine and 10% bovine calf serum (VWR) was used during live imaging processes.

For the FRAP measurements, FRAP experiments were performed on the A1R-MP Laser Scanning Confocal Microscope (Nikon) using a 100x 1.49NA oil immersion objective, which was driven by Nikon NIS-Elements software and was equipped with PMT detectors. Condensates were bleached with a solid state laser (Coherent) with a 488 nm, 100% laser intensity for 200 ms. Time-lapse images were acquired before and after photobleaching at two frames per sec for 9 loops and 82 loops, respectively. We set the bleach regions as a 1 μm diameter circle, though the region that was actually affected by the bleaching was ~1.5–2 μm in diameter.

Images were processed using FIJI to measure the intensity of the regions of interest (FRAP region, background region, and the reference region) before and after photobleaching. The selection of regions of interest and FRAP analysis were performed following the guide of the protocol of the Aravin lab and EMBL [46]. Briefly, we measured the intensity of three different regions to assess photobleaching: (1) BL: the region that was bleached by the laser, (2) BG: a region outside of the cell, used to measure the level of background signal, and (3) REF: a region inside of the cell but outside the bleached region, used to assess the decay of signal from imaging-associated photobleaching. After determining the intensities of the three regions over time, we subtracted the background signal (BG) from the BL and REF regions and normalized the fluorescence decay by dividing BL by REF to get the normalized intensity over time I(t): $I(t)=\frac{BL-BG}{REF-BG}$. Next, to calculate the fraction of fluorescence recovery after photobleaching, we divided the intensity after bleaching by the mean intensity before bleaching to get: $FRAP(t)=\frac{I(t,post-bleaching)}{I(mean\;intensity\;of\;pre-bleaching)}$. Plotting FRAP(t) over time gave an intensity curve such as that shown in (Fig 6B). Finally, we used MATLAB(R2019a) to fit the intensity curve with the equation $FRAP(t)=a*(1-e^{-bt})+c$, where a is the slowly recovering fraction; b is recovery rate constant; c is the rapidly diffusing fraction [46–49]. The fraction of mobile protein = a+c, and the fraction of immobile protein = 1-(a+c). The halftime of recovery (t_1/2_) = ln2/b.

### Bioinformatics

#### Identification of +TIPs in other organisms

A range of nine model organisms (from plants to humans) was chosen to assess the conservation of the presence of intrinsically disordered regions (IDRs) in a set of well-recognized +TIP network proteins. The selected organisms are: *Homo sapiens* (taxid:9606), *Mus musculus* (taxid:10090), *Xenopus laevis* (taxid:8355), *Danio rerio* (taxid:7955), *Drosophila melanogaster* (taxid:7227), *Saccharomyces cerevisiae* (taxid:4932), *Schizosaccharomyces pombe* (taxid:4896), *Dictyostelium discoideum* AX4 (taxid:352472), and *Arabidopsis thaliana* (taxid:3702).

To identify core +TIPs (or other relevant proteins) in these organisms, we performed a BLASTp (or when necessary, a PSI-BLAST) search in the NCBI RefSeq database using the following representative human sequences as probes: CLIP-170 (AAA35693.1), EB1 (NP_0364571), MAP215 (NP_0010089381), CLASP2 (NP_0013525571), CLASP1 (NP_0560971), p150 (NP_0040732), LIS-1 (NP_0004211), APC (NP_0013418251), and mDia2 (NP_0010359821). In many cases (e.g., EB1), identification of unambiguous homologs was straightforward from examination of the e-values and alignments; in cases where gene duplications had occurred, we chose the gene with the strongest (lowest) e-value for further analysis. In cases of alternative splicing, we chose the longest of the curated transcripts for further analysis, except for CLIP-170 and CLASP homologs, where we chose the form that best matched the domain structure of the human protein used as the probe. Note that CLIP-170 homologs were not identified in *Dictyostelium* or *Arabidopsis*.

#### Bioinformatic tools used

*IDR prediction*. We focused our efforts on three IDR predictors, which were used with default parameters unless otherwise indicated: 1) “ESpritz version 1.3” [50] (http://old.protein.bio.unipd.it/espritz/), used with prediction type as X-Ray and decision threshold as 5% false positive rate (5% FPR; chosen because the default setting [“Best Sw”–a weighted score rewarding prediction of IDRs] tends to overestimate IDRs); 2) “Interpro” (EMBL, https://www.ebi.ac.uk/interpro/), which provides as part of its standard analysis disorder predictions from the MobiDB-lite database [51]); and 3) “IUPred2”, used with “short disordered” setting, as obtained from the IUPred2A server (https://iupred2a.elte.hu/) [52,53]. We chose these three out of the wide array of possible IDR predictors because they are widely-used [54], and in our preliminary analyses they rarely generated spurious reports of coiled-coil regions as IDRs, unlike some others, e.g., PONDR. In Fig 7, we present the results of all three methods for human CLIP-170; for other analyses, we simplified the display by showing only the results from the first two predictors because the results from Espritz and IUPred2 were similar. The likelihoods of intrinsic disorder as a function of amino acid position as produced by ESpritz and IUPred2 were plotted by MATLAB(R2019a). For MobiDB-lite predictions, we manually listed all predicted IDRs as obtained from Interpro. Then, we plotted the list of IDRs with the MATLAB(R2019a) “patch” function.

*Other analyses*. Sets of orthologous protein sequences were aligned by ClustalW [55] or alternatively PASTA [56]. For most of the domain structure predictions, we used a hmmscan Pfam search with e-value 0.1 at HMMER (https://www.ebi.ac.uk/Tools/hmmer/search/phmmer) [57] (S8-S11 Figs in S5 File). The only exception was CLIP-170 and its homologs. To map the domain structure of CLIP-170 homologs, we extracted from the NCBI protein information page the location of CAP-Gly domains, serine-rich regions, and zinc-knuckle motifs. Then, we aligned the human CLIP-170 FEED domain (VEEESITKGDLE) [12] to the representative sequences of other organisms to identify the FEED domain. Lastly, we combined all the domain information and plotted the protein sequence with “IBS Version 1.0” (http://ibs.biocuckoo.org/online.php). To predict coiled-coil regions, we used the “COILS” predictor (https://embnet.vital-it.ch/software/COILS_form.html) [58] with default settings.

## Results

The overall goals of this study were to test whether the so-called “patches” induced by CLIP-170 overexpression [9,19] are biomolecular condensates and, if so, to then consider the implications of this observation for the structure and function of the +TIP network under normal physiological conditions. Although it would be ideal to examine the endogenous +TIP network directly, the “comets” at the ends of MT plus ends are both dynamic and small (near the diffraction limit of visible light), making it technically difficult to determine clearly whether they have condensate properties. Thus, although CLIP-170 patches are themselves overexpression artifacts, we hypothesized that they might reflect some properties of physiological +TIP networks and so used them as a tool to gain insight into these properties. As evidence that this approach can be fruitful, in previous work we successfully used this same logic to identify the +TIPs EB1 and dynactin complex as CLIP-170-interacting proteins and showed that the interactions were mediated by different regions of the CLIP-170 protein [19].

As noted above, four properties common to biomolecular condensates are 1) elastic deformability, 2) the ability to undergo fusion and fission, 3) selectivity: droplets include certain members but exclude others, and 4) rapid exchange of proteins between the droplets and the cytoplasm [21–23,39]. Thus, we examined the CLIP-170-induced patches for each of these characteristics in turn. In addition, proteins included in droplets generally have the following structural characteristics that are important for droplet formation: they have multiple sites for binding other droplet members (i.e., they are multivalent), and they contain intrinsically disordered regions (IDRs). Accordingly, we also examined CLIP-170 and other +TIPs for these characteristics.

### CLIP-170 induces formation of so-called "patches" upon overexpression

**Fig 2:**
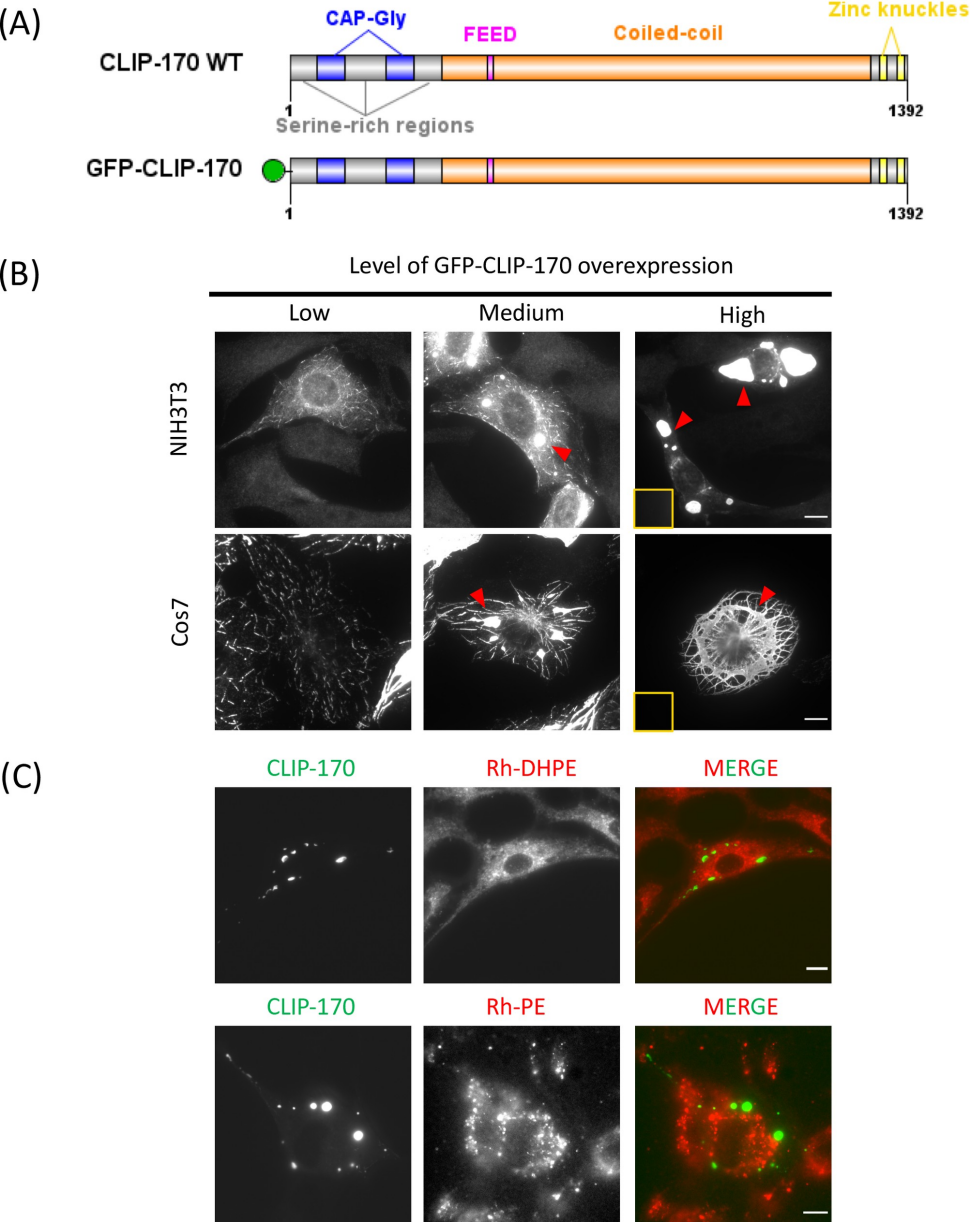
CLIP-170 patches do not colocalize with membrane markers. (A) Domain structures of the CLIP-170 constructs used in this study. (B) NIH3T3 or Cos-7 cells (as indicated) were transfected for 24 hr to overexpress GFP-CLIP-170, fixed with PFA, and observed by widefield fluorescence microscopy. Arrows point out examples of CLIP-170 patches as observed in cells at varied expression levels as indicated. The contrast of each image in a given row was adjusted to the same levels, except for high-level expression, where the main image was adjusted to allow the visualization of large and bright patch structures, and an inset provides a version normalized to match the rest of the row. (C) As an example of the failure of membrane markers to colocalize with patches, NIH3T3 cells were transfected with GFP-CLIP-170 for 24 hours. Rhodamine-DHPE or Rhodamine-PE was used as a generic membrane label to stain lipid membranes. Live cells were observed by widefield fluorescence microscopy. See S2 Fig in S5 File for colocalizations with additional membrane markers. Scale bar: 10 μm.

As shown previously, when CLIP-170 is expressed at low levels, it labels MT plus ends ([8,41,59]; see also Movies 1–2 in S1 & S2 Files), but at higher levels it causes increasing formation of patches and MT bundles (Fig 2B, [9,19]; see also Movies 3–4 in S3 & S4 Files). This phenomenon is seen in a range of cell types, including HeLa [9,19], Cos-7 ([19], Fig 2B), and NIH3T3 (Fig 2B).

This patch formation requires an intact second zinc knuckle [19]. In addition, we observed that the ability of CLIP-170 constructs to induce patches requires a free CLIP-170 C-terminus: either a GFP or a short tag of 4 amino acids at the C-terminus prevents patch formation (S1 Fig, N-18 and N-18 GSSG in S5 File). Strikingly, the ability of CLIP-170 to induce patch formation can be rescued by insertion of a stop codon before the C-terminal tag, thus reconstituting the original C-terminus (S1 Fig N-18 ETF in S5 File). These observations provide evidence that the ability of CLIP-170 to induce patches requires specific sequences and is thus not a generic effect of CLIP-170 overexpression.

### The CLIP-170-induced patches do not colocalize with membranes

To test whether the patches are membraneless, we first examined the patches for colocalization with various specific membrane markers, including MitoTracker, LysoTracker, ER tracker, GalT (labels Golgi), and EEA1 (labels endosomes). None of these, with the possible exception of EEA1, showed any significant colocalization with the CLIP-170 patches (S2 Fig in S5 File).

One problem with using specific makers to test for membrane presence is that cells contain many different membrane-bound compartments, and so negative results are not definitive: we might simply have failed to test for the appropriate compartment. Therefore, we tested for the presence of membranes more generally by looking for colocalization with the nonspecific membrane dyes Rhodamine-DHPE and Rhodamine-PE. We observed no obvious colocalization with either dye (Fig 2C). These results are consistent with the hypothesis that the CLIP-170-induced patches are membraneless biomolecular condensates. Therefore, we proceeded to test for the presence of other characteristics of biomolecular condensates.

### CLIP-170 patches can elastically deform and undergo fusion and fission

**Fig 3:**
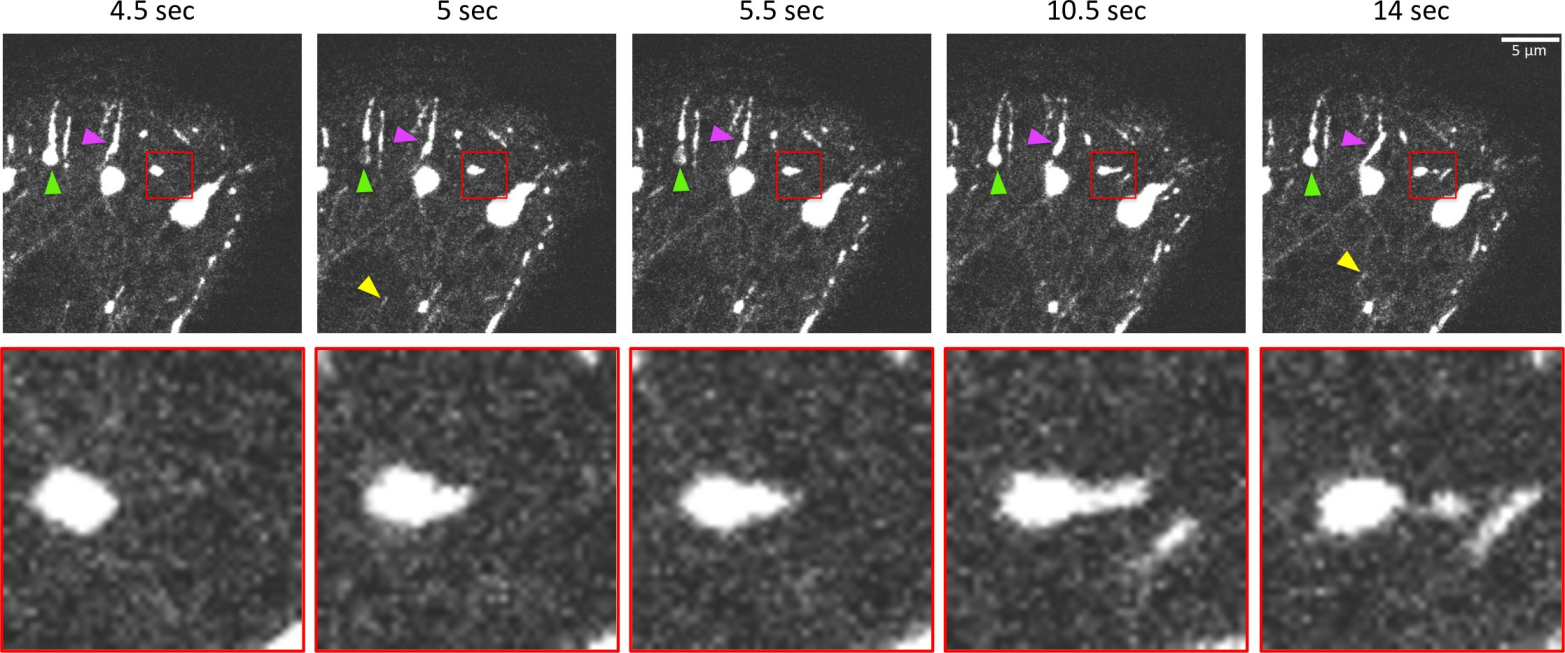
Dynamic behaviors of CLIP-170 patches *in vivo*. NIH3T3 cells were transfected to overexpress GFP-CLIP-170, and the behavior of GFP-CLIP-170 *in vivo* was recorded by confocal microscopy after 24–27 hr of transfection. Red box: An example of an apparent elastic deformation of a patch, following by fission (blow-up of this region is shown below). Arrows indicate examples of patch fusion (magenta), a photobleached site (green, relevant to discussions below), and a typical comet-shaped +TIP localization on a MT tip (yellow).

As discussed above, another characteristic of biomolecular condensates is that they can undergo elastic deformations, fission, and fusion. To test whether CLIP-170 patches exhibit these activities, we overexpressed GFP-CLIP-170 in NIH3T3 cells and used live cell fluorescence microscopy to monitor the GFP-CLIP-170 behavior over time. As shown in Fig 3 and Movie 3 in S3 File, CLIP-170 patches do indeed display fusion events (magenta arrow) and fission events (red box). In addition, it is common to see CLIP-170-labeled +TIP comets travel through and deform the CLIP-170 patches (Fig 3 and Movies 3–4 in S3 and S4 Files). This observation indicates that the CLIP-170 patches interact with the +TIP network and suggests that these two structures may share some properties with each other. Taken together, these results indicate that the CLIP-170 patches have the elastic deformability, fusion, and fission properties expected of a biomolecular condensate.

### CLIP-170 patches selectively recruit some cytoskeletal proteins but exclude members of other biomolecular condensates

The next biomolecular condensate property we tested was selectivity: do CLIP-170 patches selectively recruit some proteins and exclude others? To address this question, we used immunofluorescence staining to test various molecules for patch colocalization. The two groups of markers we tested were: (1) members of the +TIP network and (2) proteins included in established cytosolic liquid droplets (Table 1).

**Table 1:** List of molecules we have tested for co-localization with CLIP-170 patches.

Protein	Included/excluded	Protein marker of
**EB1**	Included	+TIPs
**LIS-1**	Included	+TIPs
**APC**	NSAI	+TIPs and Wnt signaling
**CLASP1**	Included	+TIPs
**CLASP2**	Included	+TIPs and Golgi
**MAP215**	NSAI	+TIPs
**p150**	Included	+TIPs and Dynactin
**mDia2**	Included	Formin
**RNA**	Excluded	RNP granules
**YB1**	Excluded	Stress granules and P-bodies
**hnRNPA1**	Excluded	RNP granules
**Staufen1**	Excluded	RNP granules
**Tubulin**	Included	

**Fig 4:**
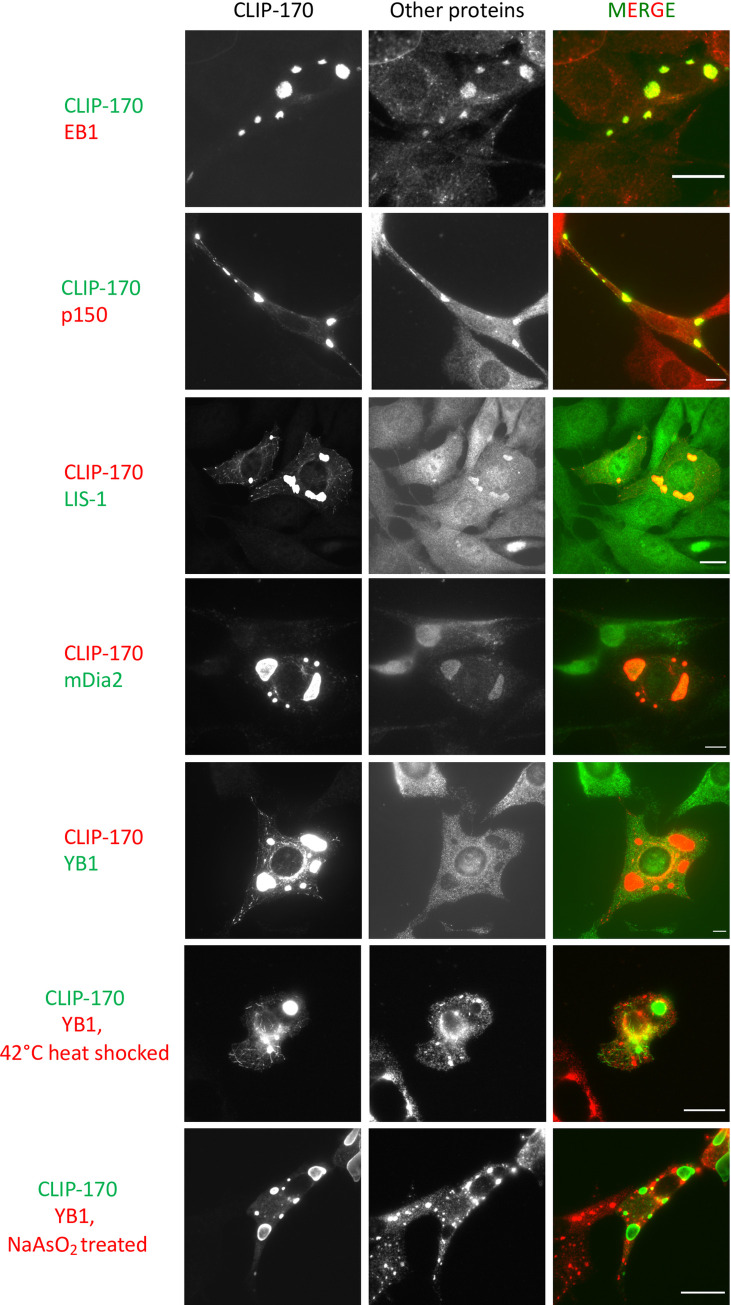
CLIP-170 patches have selective properties. NIH3T3 cells were transfected with full-length CLIP-170 for 24 hr before fixation and staining as indicated. For the EB1, p150, and YB1 labeling, cells were fixed with methanol; for LIS1 and mDia2, cells were fixed with PFA. The contrast of all images in a given row was set to the same levels. These data show that EB1, p150, LIS-1, and mDia2 were included in the CLIP-170 patches; YB1 is an example of a molecule that was excluded from the patches. See S3 Fig in S5 File for images of colocalizations with other molecules, and see Table 1 for a summarized list. Scale bar: 10 μm.

Experiments were conducted in NIH3T3 cells. NSAI indicates “No Suitable Antibody Identified” due to lack of suitable antibodies. See Fig 4 and S3 Fig in S5 File for images of the colocalizations summarized above.

As expected from previous work [9,19], the +TIPs EB1 and p150 dynactin were observed to be recruited to the CLIP-170 patches (Fig 4, Table 1). In addition, we found that the patches recruit the +TIPs LIS1, CLASP1, CLASP2, as well as mDia2, an actin nucleator that binds EB1 [60] and so might be described as a peripheral part of the +TIP network (Fig 4, S3 Fig in S5 File, and Table 1). However, antibodies against at least one other protein that has been described as MT binding (Staufen1) did not colocalize with CLIP-170 patches (S3 Fig in S5 File, Table 1).

To test for possible overlap between the CLIP-170 patches and other biomolecular condensates, we checked markers of RNP granules, P-bodies, and stress granules for colocalization to the patches. None were included in the CLIP-170 patches under our normal culture conditions (Fig 4, S3 Fig in S5 File, and Table 1). Because stress granules are typically visible only under stress, we also tested colocalization with the stress granule protein YB1 after 42°C heat shock or 0.5 mM NaAsO_2_ (a classical stress inducer for YB1 containing stress granules [61]); we also examined Staufen1 after 42°C heat shock. The heat shock did induce the formation of structures containing these proteins, but they did not colocalize with the CLIP-170 patches (Fig 4, S3 Fig in S5 File, and Table 1).

**Fig 5:**
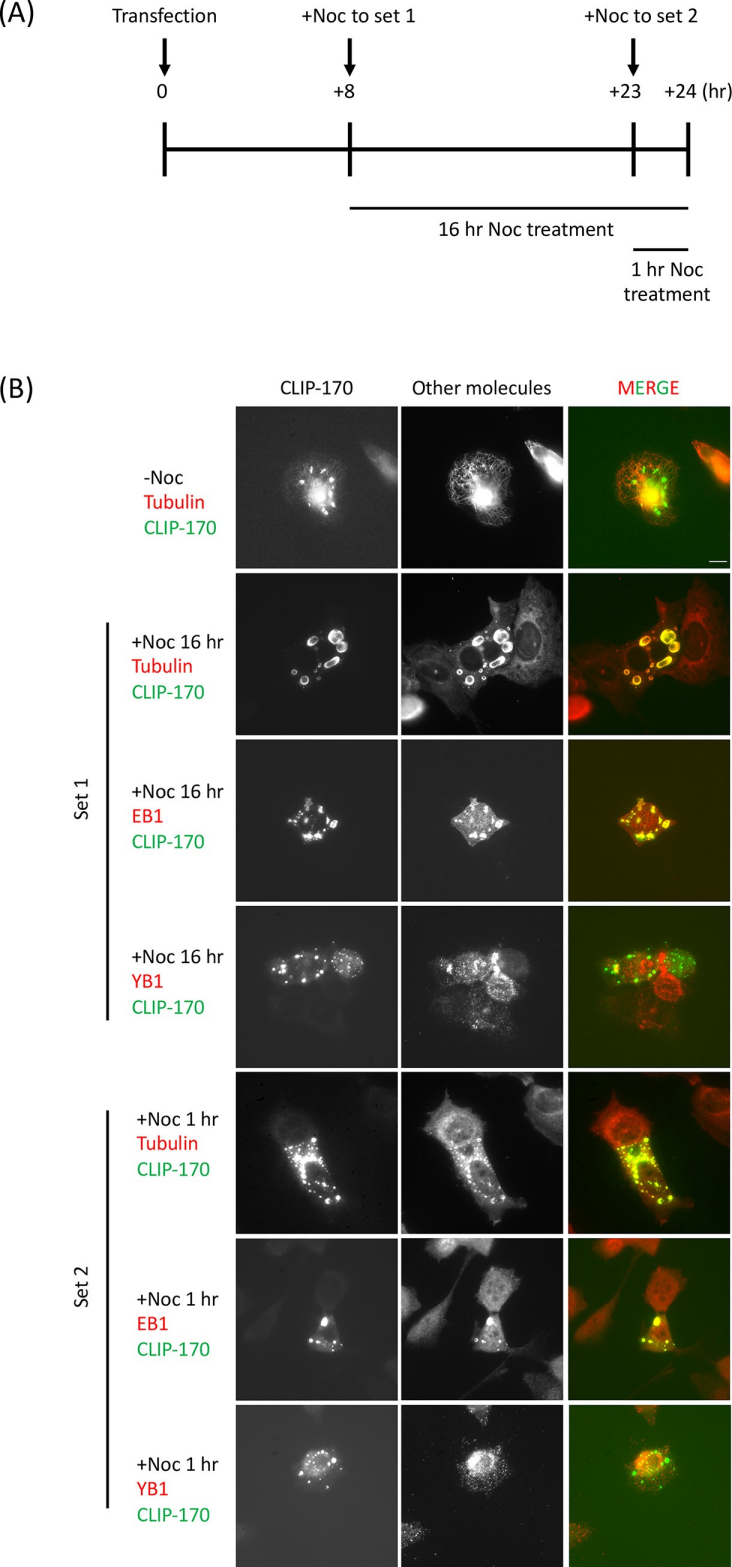
MTs are not required for CLIP-170 patch formation and maintenance. (A) Experimental design diagram. (B) NIH3T3 cells were transfected with full-length CLIP-170, and cells were treated with 5 μM nocodazole (Noc) after 8 hr (Set 1) or 23 hr (Set 2) transfection at 37°C. Cells were fixed with methanol after 24 hr transfection, probed with antibodies against molecules of interest (as indicated), and observed by widefield fluorescence microscopy. After the nocodazole treatment, tubulin and EB1 colocalized with CLIP-170 patches, while YB1 did not colocalize with the patches. Each image was normalized separately to allow the visualization of the different staining and patches. Scale bar: 10 μm.

One question not addressed above is whether the patches colocalize with MTs and/or tubulin. We observed that colocalization of the patches with MTs was variable. Staining in methanol-fixed cells under normal cell culture conditions typically revealed a tangle of MTs colocalized with the patches (Fig 5B, top row, see also [19]). However, in PFA-fixed cells, the tubulin staining was sometimes limited to the outside of the patch (S4 Fig in S5 File, see also [9]).

The colocalization between patches, MT-binding proteins (Fig 4 and S3 Fig in S5 File) and MTs (Fig 5B, top row and S4 Fig in S5 File) as discussed above suggests a functional relationship with MTs. Therefore, we examined the effect of nocodazole on colocalization between patches and anti-tubulin antibodies. In setting up this experiment, we had two considerations. First, we wanted to see whether the patches are maintained in nocodazole-treated cells. Second, we wanted to see if patch formation requires MTs. Therefore, we transfected cells grown on coverslips with CLIP-170 and performed nocodazole treatments at two timepoints, as diagrammed in Fig 5A.

For the first time point (Fig 5A, Set 1), we treated some of the coverslips with nocodazole after eight hours of transfection, which is early in the transfection process and thus before significant expression of plasmid-based CLIP-170 or patch formation would be expected. We then continued to incubate these coverslips in nocodazole and fixed and imaged them at the end of the experiment, i.e., after 24 hours of transfection. For the second time point (Fig 5A, Set 2), we treated some coverslips with nocodazole after 23 hours of transfection, a time when patches are prominent in many transfected cells. We then fixed and imaged these coverslips at the same time as the coverslips from the first time point, i.e., after the same 24 hours of transfection.

In this experiment (results provided in Fig 5B), we observed that MTs had disassembled in both sets of nocodazole-treated cells, as expected. Significantly, we also found that patches were observed in both sets of nocodazole-treated cells. Consistent with earlier results showing that EB1 is recruited to the CLIP-170 patches (Fig 4 and [19]), the CLIP-170 patches in nocodazole-treated cells also contained EB1. In addition, the stress-granule marker YB1 was still excluded from patches in nocodazole-treated cells, even though nocodazole induced the formation of stress granules (Fig 5B).

The observation that patches are observed in both sets of nocodazole-treated coverslips (Fig 5B, Set 1 and Set 2) indicates that MTs are not required for patch maintenance. However, we cannot rule out the possibility that small numbers of nocodazole-stable MTs are sufficient for patch maintenance. The observation that patches were observed at the end of the experiment in Set 1 initially suggested that MTs are not needed for patch formation, since (as noted above), we expected that no cells would have patches when these cells were treated with nocodazole (i.e., after eight hours of transfection). However, to our surprise, we found that some transfected cells did have patches when fixed at this 8 hr time point (S5 Fig in S5 File). Therefore, whether patch formation requires MTs remains a question for further study. Regardless, in both sets of nocodazole-treated cells, tubulin was clearly recruited to the patches, as evidenced by the bright tubulin staining (Fig 5).

Taken together, these data demonstrate that CLIP-170 patches selectively include a specific set of +TIP network proteins as well as tubulin (at least in nocodazole), while excluding members of several other known biomolecular condensates. This selective property is again consistent with the idea that the patches are biomolecular condensates. Furthermore, the observation that known condensate markers are excluded from the patches suggests that they are novel biomolecular condensates.

### CLIP-170 in the patches exchanges rapidly within the cytoplasm

**Fig 6:**
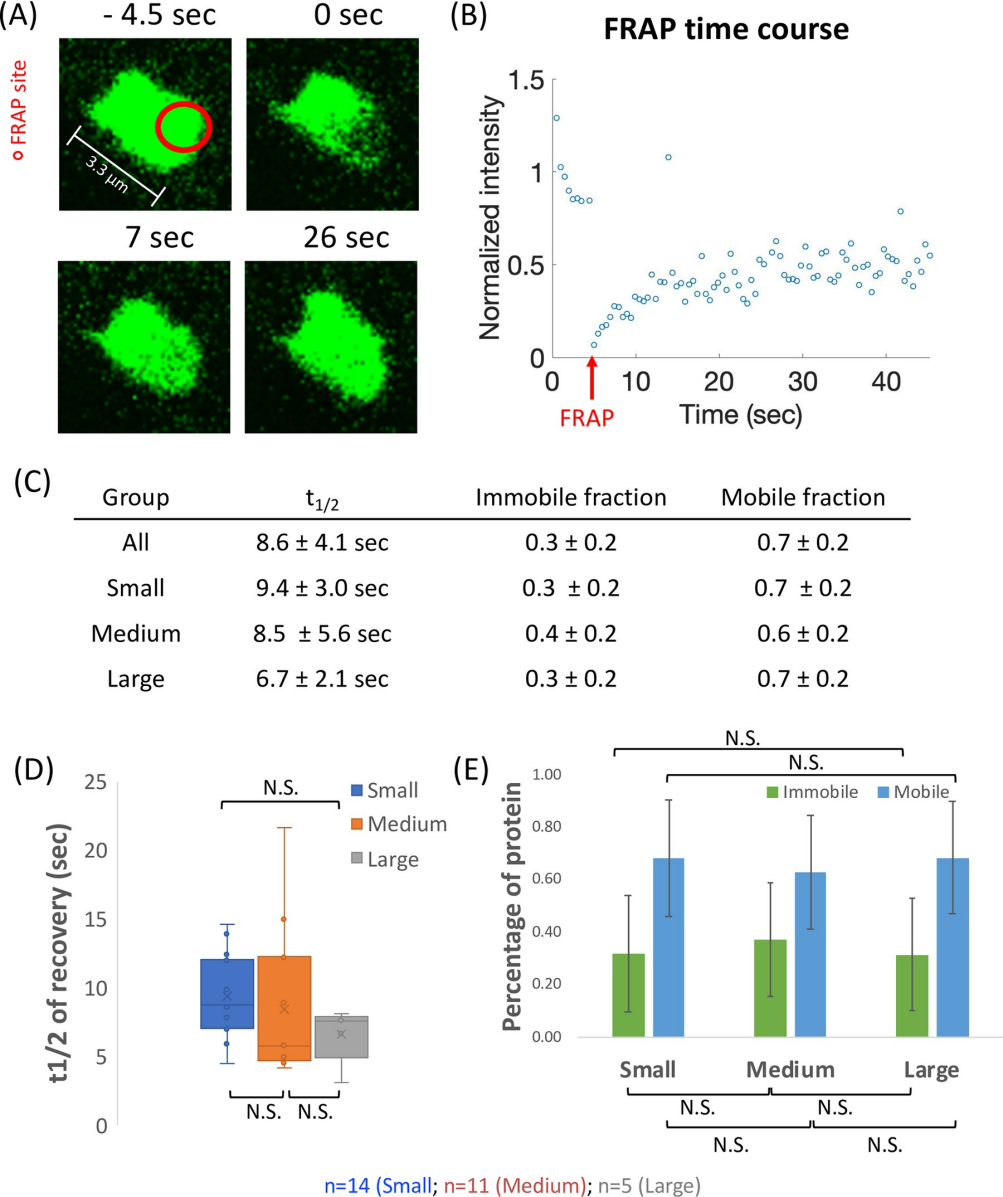
FRAP analysis of the dynamics of CLIP-170 patches. (A) Example of a partially photobleached CLIP-170 patch in an NIH3T3 cell. (B) The time course plot for the FRAP spot in (A). This panel is one example representing the fluorescence recovery of one bleaching site in one CLIP-170 patch to show how we processed the data. The intensity values were normalized against the average mean intensity before bleaching. (C, D, E) Quantification of the t_1/2_, mobile fraction, and immobile fraction. A total of 30 condensates from 30 separate cells were photobleached, and their recovery profiles were fitted to the exponential recovery equation (see Methods for details). “All” indicates the summation of data without regard to droplet size. In addition, we analyzed the droplets as separated into small, medium, and large groups (see S6 Fig in S5 File for more information about how this separation was performed). (C) provides a numerical summary of the t_1/2_, mobile fraction, and immobile fraction. Panel (D) provides a box plot of the t_1/2_ of each size group, while panel (E) provides a bar graph of the fractions of mobile and immobile protein. n = 14 (Small), 11 (Medium), and 5 (Large), for a total of 30 droplets. The error bars show the standard deviation; other aspects of the box plot are as generated by the default Excel Box and Whisker plot function. N.S. = Not Significant (there were no significant differences between any of these groups).

Another expected characteristic of a biomolecular condensate is the rapid molecular exchange of molecules between the condensate and the cytoplasm. A technique commonly used to assess such exchange is Fluorescence Recovery After Photobleaching (FRAP). We performed FRAP assays on the CLIP-170 patches and observed that the fluorescence intensity of the photobleached spots recovered quickly (half time of recovery (t_1/2_) = 8.59 ± 4.05 sec) and to a large degree (0.66 ± 0.21 mobile fraction) (see example in Fig 6). This rate and degree of recovery of CLIP-170 patches is consistent with those reported for other biomolecular condensates [62].

To further understand the kinetics of CLIP-170 patches, we categorized our FRAP data into three groups by patch diameter to determine whether the fluorescence recovery time and/or degree varied with size: small (0–2.4 μm), medium (2.4–4.4 μm), and large (≥ 4.4 μm). The grouping strategy is based on the size distribution after measuring the diameters of ~300 condensates (S6 Fig in S5 File).

We observed that while the recovery of the medium patches was more variable than that of large and small patches, there was no significant difference between the groups in either the recovery rate or recovery extent (Fig 6C–6E). These observations are notable for three reasons. First, since bleaching generally covered the full area of the small patches, the observation that small patches recover quickly confirms that the patches are not membrane bound because the interior of a membrane-bound compartment would not be expected to exchange material with the cytoplasm (exterior) on this time scale. Second, the observation that all three patch sizes recover with a similar rate indicates that the surface area: volume ratio is not rate limiting for material exchange with the cytoplasm. This in turn suggests that the patches are relatively porous. Third, the observation that the size of the condensates does not obviously correlate with the observed exchange dynamics is consistent with the idea that the CLIP-170 patches may reflect processes occurring in the small, endogenous +TIP network comets (~0.5 μm).

Taken together, our results thus far demonstrate that the CLIP-170 patches share key properties with known biomolecular condensates: they are membraneless, they undergo elastic deformations, fission, and fusion, they selectively include some molecules (e.g., +TIPs) while excluding others (e.g, stress granule proteins), and proteins exchange rapidly between patches and the cytoplasm. These observations lead us to conclude that the CLIP-170-induced patches are biomolecular condensates.

### Sequence information is consistent with the idea that CLIP-170 is a component of biomolecular condensates

If CLIP-170 is inducing biomolecular condensates, one would expect CLIP-170 to have sequence attributes consistent with this activity. As mentioned above, proteins found in condensates (i.e., proteins that have the potential to undergo phase-phase separation) typically are multivalent (i.e., have multiple binding sites for other biomolecules) and contain intrinsically disordered regions (IDRs). Indeed, it is already well-established in the literature that CLIP-170 has multivalent interactions with multiple other proteins: a dimer of CLIP-170 has as many as ten binding sites for tubulin [15], multiple binding sites for EB1 [16], and an array of binding sites for other proteins including p150 (reviewed by [3]). However, while it seems widely known that CLIP-170 contains IDRs, we were not able to find a formal analysis of this question in the literature.

**Fig 7:**
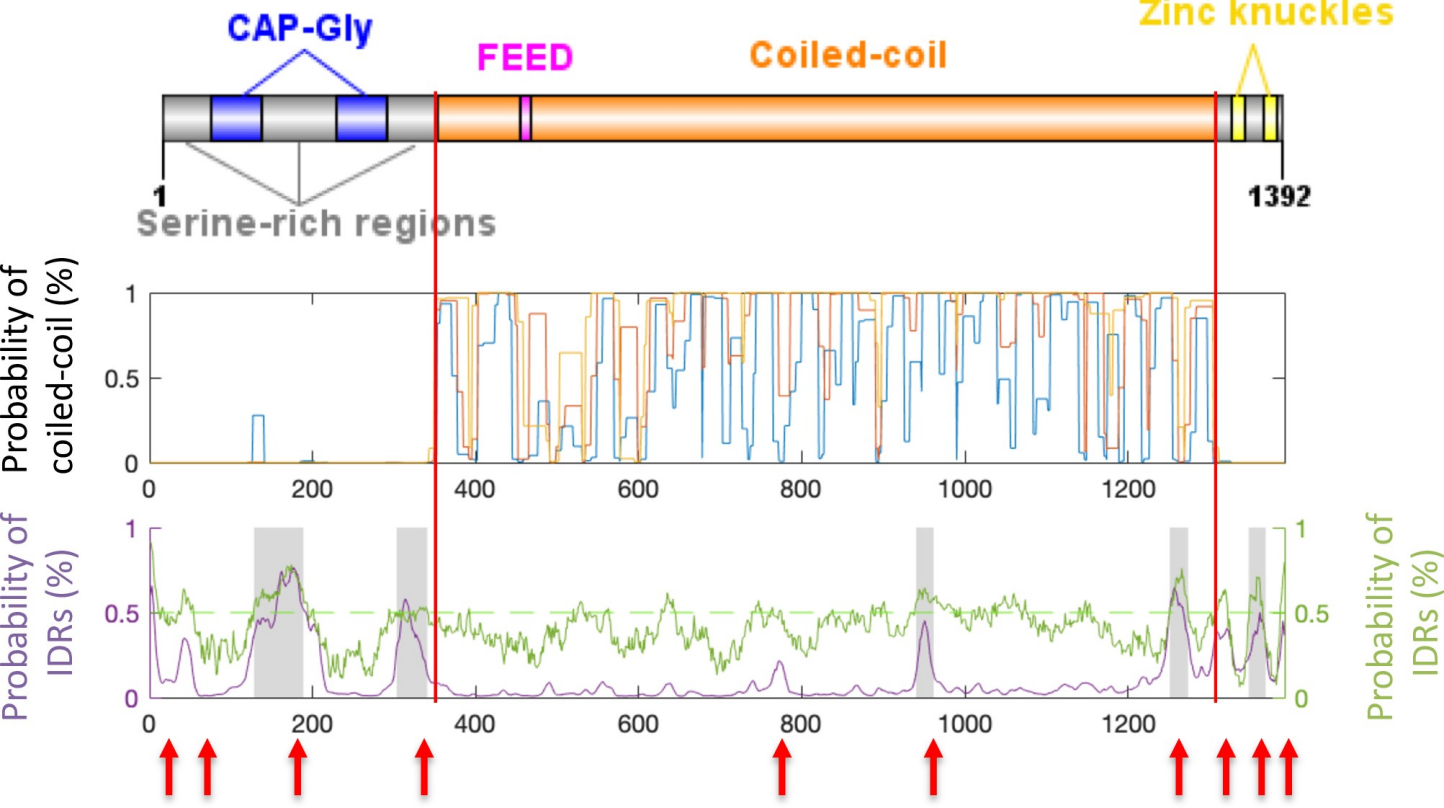
Analysis of coiled-coil domains and IDRs for human CLIP-170 (AAA35693.1). (Top) CLIP-170 domain structure summary. The N-terminal domain includes the 2 CAP-Gly domains and 3 serine-rich regions. The coiled-coil domain is labeled in orange, and its boundaries are indicated with two red lines. The C-terminal domain contains two zinc-knuckle motifs (yellow). (Middle) Analysis of coiled-coil propensity as predicted by COILS. The colored lines represent the probability that a region assumes a coiled-coil conformation, as assessed for different windows (7AA, blue; 14 AA, orange; 21 AA yellow), with 1 on the Y-axis indicating 100% likelihood. (Bottom) IDR predictions (Y-axis) as function of amino acid position (X-axis), with 1 indicating 100% likelihood. The purple line represents the probability of IDRs as predicted by Espritz, with red arrows indicating the 10 regions predicted to be disordered. The grey areas indicate the IDRs as predicted by MobiDB-lite. The green line represents the probability of IDRs as predicted by IUPred2A, and the light green indicates the probability of 0.5.

Therefore, we evaluated the sequence of human CLIP-170 for the presence of disordered regions by using the established predictors “ESpritz version 1.3” [50], “MobiDB-lite” [51], and “IUPred” [52]. In parallel, we used the “COILS” server to delineate the coiled-coil region (Fig 7) because we wanted to focus on IDR predictions outside of likely coiled-coil; this was important because in preliminary work, some predictors (e.g.,PONDR) frequently identified coiled-coil regions as likely IDRs (see also [63]). These analyses show that the three serine-rich regions in the N-terminal domain and regions adjacent to the zinc-knuckles in the C-terminal domain are predicted to be disordered in human CLIP-170 (Fig 7).

**Fig 8:**
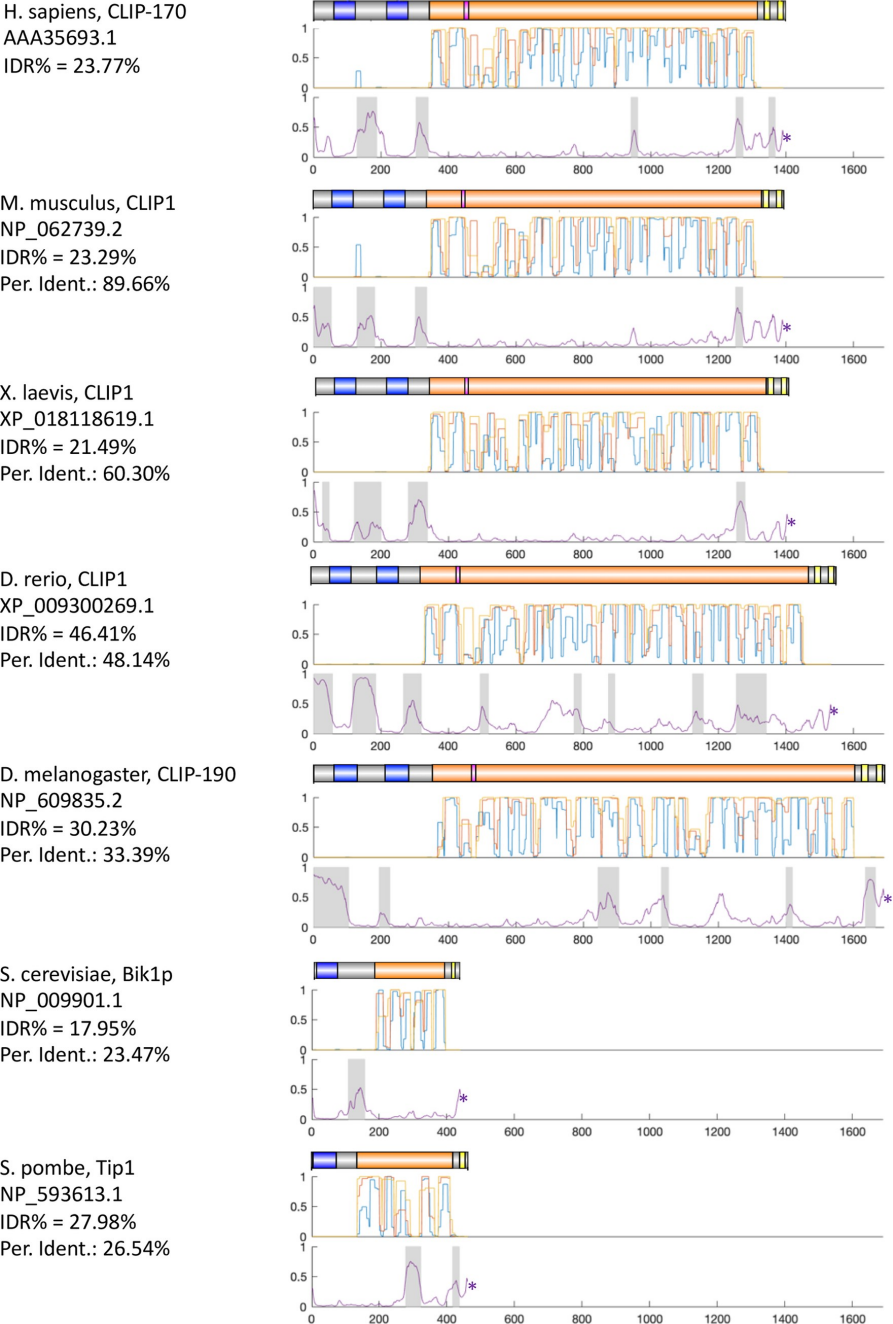
Analysis of coiled-coil and IDRs in CLIP-170 relatives from a range of different species. For each organism listed, one representative CLIP-170 sequence was selected for analysis, the results of which are shown in three images. The set of upper images provides the domain structure for each sequence, with colors as indicated: blue: CAP-Gly domains; orange: Coiled-coil regions; pink: FEED domain; yellow: Zinc-knuckle motifs. The sets of middle and lower images respectively provide the probabilities that a region is coiled-coil (as predicted by COILS) or IDR (as predicted by Espritz). For both analyses, 1 indicates 100% likelihood and * indicates the end of the sequence.

The observations that CLIP-170 is multivalent [3,15,16] and contains IDRs (Fig 7) are consistent with the idea that CLIP-170 can participate in biomolecular condensates. If these attributes are functionally significant, they should be conserved in diverse organisms. Indeed, examination of CLIP-170 relatives shows that the domain structure of CLIP-170 is conserved across diverse organisms (Fig 8 and S7 Fig in S5 File), as previously noted in many publications; conservation of the domain structure implies conservation of multivalency. Moreover, our analysis demonstrates that the presence of IDRs adjacent to the CAP-Gly motifs and zinc knuckles is also well-conserved, extending even to the yeasts *S*. *cerevisiae* and *S*. *pombe* (Fig 8 and S7 Fig in S5 File).

Two additional points are relevant to interpreting these data. First, though IDRs are not universally detected in the same places in all the organisms shown, assignment of IDRs is still an imperfect process; it remains possible that some of the proteins that appear to lack IDRs in particular regions (e.g., serine-rich 3 of Drosophila, the post-CAP-Gly serine-rich region of *S*. *pombe*) actually have them. Second, IDRs are sometimes identified in the coiled-coil regions, consistent with the established propensity of IDR prediction programs to find predicted IDRs in coiled-coil [63]. The observation that these predicted IDRs are less conserved in their positions than are the IDRs near the CAP-Gly domains and zinc knuckles suggests that these may be spurious assignments.

In conclusion, the sequence characteristics of CLIP-170 and its relatives are consistent with the idea that CLIP-170 participates in biomolecular condensates and that this activity is conserved across a wide range of organisms.

### IDRs in other +TIP proteins are both common and conserved

Thus far, our analyses have focused on CLIP-170 and the condensates induced by CLIP-170 overexpression. As discussed in the Introduction, we are interested in the possibility that these large induced condensates reflect a much smaller +TIP network structure that exists under normal conditions, on the MT +TIP and/or in other cellular locations. Our sequence analyses (Fig 8 and S7 Fig in S5 File) suggest that the ability of CLIP-170 to participate in condensates is likely to be conserved from humans to yeast, consistent with the idea that condensate formation is functionally significant. However, CLIP-170 can be deleted from some organisms (e.g., *S*. *cerevisiae*) with relatively minor effects (S1 Table in S5 File), and other organisms (e.g., *Dictyostelium* amoebas, plants) lack CLIP-170 entirely, arguing against a central role for CLIP-170 in function of the +TIP network.

**Fig 9:**
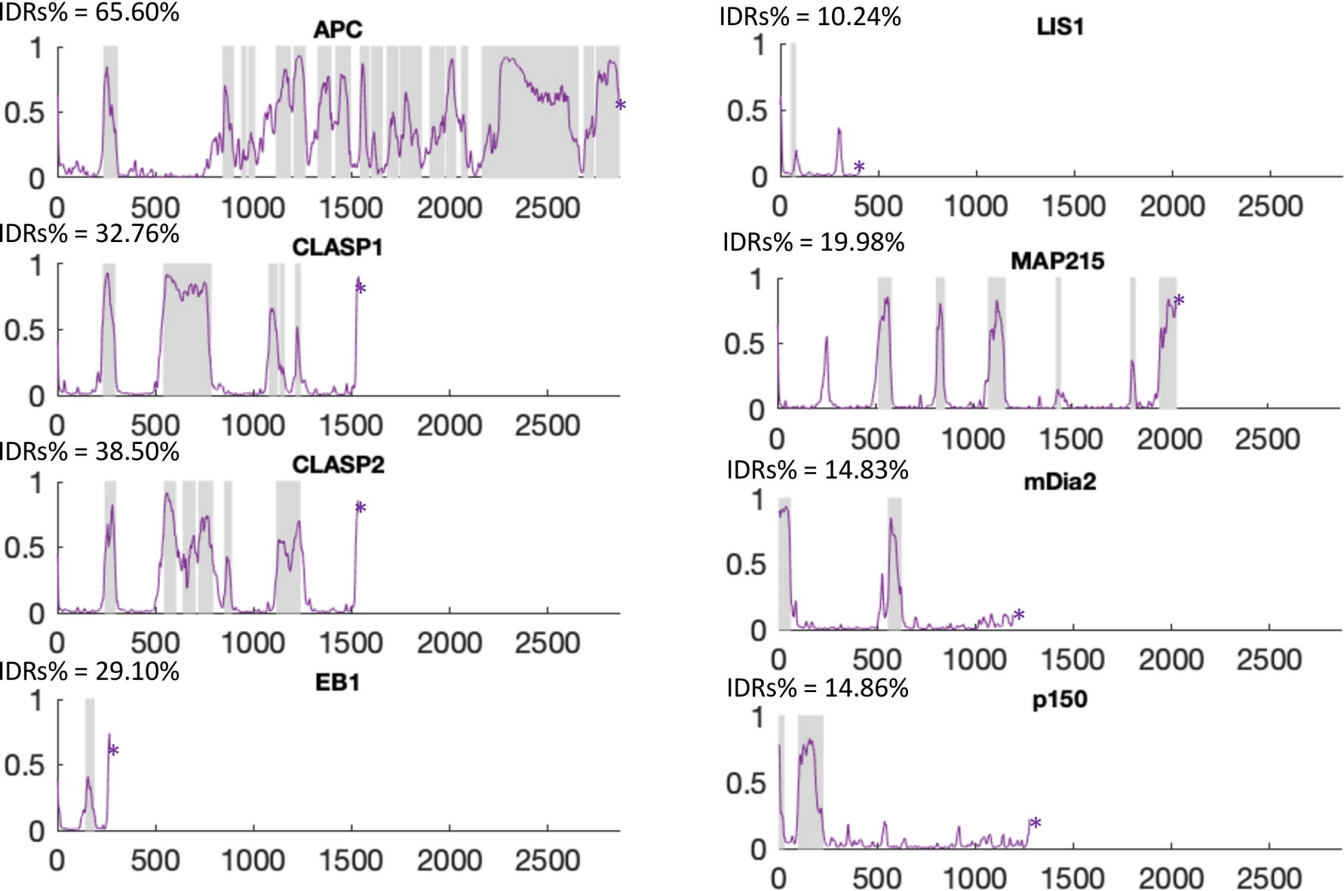
Analyses of IDRs in members of the human +TIP network. The Y-axis provides the probability of IDRs as a function of amino acid position (X-axis); 1 indicates 100% likelihood. The purple line provides the data for Espritz; shaded areas are the disordered regions predicted by MobiDB-lite. See Materials and Methods for the accession numbers.

These observations indicate that if the normal activities of the +TIP network involve a functionally significant biomolecular condensate, other +TIPs should be able to drive condensate formation. Addressing this question in depth is beyond the scope of this paper. However, as a first step, we examined the sequences of other +TIPs to see if they have characteristics consistent with condensate formation, i.e., multivalency and the presence of IDRs. As noted above, it is already well-established that many human +TIPs are multivalent [3]. Therefore, we examined the sequences of other human +TIPs to see if they contain IDRs. As part of this analysis, we included other proteins that colocalized with CLIP-170 from our immunofluorescence study (Fig 9).

Our analyses show that the human versions of proteins that colocalized with CLIP-170 patches (EB1, LIS1, CLASP1, CLASP2, mDia2, and p150) contain predicted IDRs, as do other common +TIPs (APC and MAP215). As part of this work, we also examined sequences for the presence of alpha helical coiled-coil (using the COILS server) and the other conserved domains (using hmmscan to search the PFAM database). Comparison of these results with the IDR predictions shows that the predicted disordered regions are mostly outside of alpha-helical coiled-coil and recognized folded domains (e.g. the CH domain in EB1) (S8 Fig in S5 File), consistent with the idea that the IDR predictions are accurate. Although prediction of IDRs from sequence information is still maturing, and though presence of IDRs in a protein does not necessarily indicate that the protein participates in condensate formation, our results show that IDRs are very common in proteins involved in the +TIP network.

If the IDRs are functionally significant, their presence and perhaps also their positions relative to other conserved domains should be conserved across divergent organisms. To test these predictions, we chose the three most ubiquitous and conserved +TIPs (EB1, MAP215, and CLASPs) and examined their sequences for the presence of IDRs and recognized domains in organisms ranging from humans to plants. The results (S9-S11 Figs in S5 File) show that both the presence and position of disordered regions is well-conserved in all three proteins throughout all of the species examined.

More specifically, in EB1 (S9 Fig in S5 File), both MobiDB-lite and Espritz predict a disordered region for all species examined upstream of the coiled-coiled region, between the CH and EBH domains. The Espritz predictor also predicts a disordered region after the EBH domain for all species (S9 Fig in S5 File). In MAP215, both predictors find disordered regions outside of the TOG regions. The pattern of disordered regions is surprisingly similar for MAP215 in all species, except in yeast, where the length of the MAP215 homologs is dramatically reduced (S10 Fig in S5 File). In CLASP, the presence and position of disordered regions are highly conserved from human to fly. The similarity of the domain structures is less obvious in yeast, *Dictyostelium*, and plants, but it is notable that the CLASP homologs in all of these organisms still contain IDRs (S11 Fig in S5 File).

In conclusion, analysis of the protein sequences demonstrates that CLIP-170 and other members of the +TIP network have IDRs, consistent with participation of these proteins in biomolecular condensates. Moreover, the presence and position of these IDRs is well-conserved across animals and (where relevant) to organisms as divergent as plants, indicating that these IDRs are functionally significant. Moreover, the observation that the general domain structure of key +TIPs is conserved in organisms as diverse as plants (Fig 8 and S9-S11 Figs in S5 File) indicates that multivalency is also likely to be a conserved feature. These observations are consistent with the hypothesis that the +TIP network can assemble into a functionally significant biomolecular condensate.

## Discussion

### The “patches” induced by CLIP-170 are biomolecular condensates

Our results demonstrate that the so-called “patches” induced by CLIP-170 overexpression [8,19] have the key hallmarks of biomolecular condensates (i.e., structures formed by liquid-liquid phase separation): elastic deformability (Fig 3 and Movies 3–4 in S3 and S4 Files), the ability to undergo fission and fusion (Fig 3 and Movies 3–4 in S3 and S4 Files), selective inclusion of some proteins and exclusions of others (Figs 4 and 5, S3 and S4 Figs in S5 File, and Table 1), and rapid protein exchange with the cytoplasm (Fig 6). These observations lead us to conclude that the patches are biomolecular condensates. Significantly, the lack of overlap with established condensate markers (Fig 4 and S3 Fig in S5 File) also suggests that the patches may represent a previously unrecognized type of condensate.

While these observations indicate that the CLIP-170-induced structures are biomolecular condensates, they do not address the question of functional significance. One possibility is that the overexpression-induced +TIP condensates are simply overexpression artifacts (albeit ones based on specific interactions) and so have no functional significance. Alternatively, the +TIP condensates observed in overexpression experiments could reflect +TIP superstructures that are formed by the endogenous +TIP network. In this regard, it is significant that the ability of overexpressed CLIP-170 to drive condensate formation depends on the protein having both an intact second zinc knuckle domain (See Fig 4 in [19]) as well as a free C-terminus (S1 Fig in S5 File). These observations indicate that condensate formation is specific and is not a generic outcome of protein overexpression, but they do not directly address the question of physiological significance.

Resolving this question would be challenging, in part because the comet at the end of MTs in unperturbed cells is both small (near the diffraction limit in size) and very dynamic. Moreover, a functionally significant +TIP condensate could form in environments other than the plus-end comet (e.g., at the cell cortex). For these reasons, directly testing the hypothesis that +TIP condensate formation is functionally relevant is beyond the scope of this work. However, as a first step in this direction, we examined CLIP-170 and other +TIPs for the presence of sequence characteristics (specifically multivalency and the presence of IDRs) consistent with participation in condensates. We then investigated how conserved these characteristics are across evolution. Finding out whether condensate-associated sequence characteristics are common in human +TIP network proteins was intended to answer the question of whether condensate participation is a general feature +TIP network proteins or more specific to CLIP-170. The conservation analysis was performed as a test of functional significance, since a feature that impacts function of a basic cell biological process would be expected to be maintained across evolution.

As known from previous work [3,15,16], multivalency is indeed common in +TIPs (see also Fig 8 and S9-S11 Figs in S5 File). Importantly, conservation of the basic domain structure of these proteins across wide spans of evolution (Fig 8 and S9-S11 Figs in S5 File) indicates that multivalency is also a conserved feature of these proteins. In addition, we observed that IDRs are common in +TIPs (Fig 9 and S8 Fig in S5 File). Significantly, for key +TIPs that can be recognized in divergent organisms, the existence and position of these predicted IDRs is also well-conserved across wide spans of evolution (e.g. humans through plants, Fig 8 and S9-S11 Figs in S5 File), suggesting that these domains have activities that are functionally significant.

In considering these observations, it is important to stress that it is the existence and position of the predicted IDRs relative to conserved domains that are conserved, not the linear sequence of these predicted IDR-domains themselves. Indeed, at a primary sequence level, the regions identified as IDRs typically align poorly, once organisms outside of animals are compared (e.g., S7 Fig in S5 File). Although one might be tempted to conclude that the poor alignability of these regions suggests lack of functional significance, it is instead consistent with the idea that these regions of the +TIPs are intrinsically disordered [64]. In conclusion, our sequence analysis indicates that multiple members of the +TIP network have sequence features consistent with condensate formation and that these features are largely conserved across evolution, providing evidence that +TIP condensate formation is functionally significant.

One important question raised by the observations above is whether overexpression of other +TIPs causes condensate formation. We are not aware of other reports in the literature where overexpression of +TIPs has led to formation of structures characterized to be or described as condensates or liquid droplets. Significantly, in our hands, overexpression of EB1 (tagged at either end or untagged) does not lead to patch formation [19]. However, some reports of condensate formation by other +TIPs have appeared in unpublished meeting abstracts. Most strikingly, overexpression of full-length APC has been described as causing formation of "granules" or "aggregates" that dynamically track growing MT plus ends but differ in appearance from typical +TIP comets; these structures exhibit behaviors that to us are strongly suggestive of liquid condensates (see e.g., Fig 6 and Fig 6A–6C videos in [65]). Thus, there is reason to expect that other +TIPs can promote condensate formation as well.

So, starting from the recognition that we do not yet know whether +TIP condensate formation is physiologically significant, how *might* it be physiologically significant? As discussed more below, we propose that condensate formation may be fundamental to the core function of the +TIP network in regulating MT dynamics. As a foundation for discussing this hypothesis, we first review established functions for condensates and briefly review what is known about the mechanism of MT dynamic instability and its regulation by MT binding proteins.

### Functions of established biomolecular condensates

Before considering how a +TIP condensate might function, it is useful to first examine functions of other condensates. As discussed in the Introduction, the function of condensates is typically less clear than their existence. However, two commonly proposed functions are concentrating biomolecules to promote reactions (e.g., [37]) and segregating biomolecules into storage depots to prevent unwanted reactions (e.g, [25]). Both functions involve using condensates to alter the concentrations of reactants and thus the rates or timing of reactions. A very different set of proposed functions involves the ability of condensates to resist mechanical force [38] or even generate it [66]. Though sometimes left out of discussions of condensate function, is not surprising that assembly of condensates (which could be considered a type of 3D polymerization) would be able to generate mechanical force, given the well-demonstrated ability of actin and tubulin polymerization to generate force [67,68]. Finally, another set of proposed functions for condensates, one which overlaps with the ideas above, is that they play a role in signal transduction [69]. Indeed, one could imagine that a phase separated droplet, a local region of high concentration that is in communication with the rest of the cell, could be an ideal structure for integrating information from multiple pathways, helping the cell to produce a coherent response to conflicting signals [70].

While it is now well-known that condensates are important for processes such as stress response and RNA processing, recent work also indicates that condensate formation plays a fundamental role in cytoskeletal processes. For example, phase separation appears to play an important role in regulation of actin nucleation [71–73]. In the MT cytoskeleton, there is evidence that condensates are important to both the normal function of the MT binding protein Tau and its role in neurodegenerative disease [34–36]. Moreover, one of the most striking existing examples of functionally significant cytoplasmic condensates is the centrosome, where condensate formation helps to both concentrate and localize the MT nucleating machinery and resist the forces generated during cell division [38]. Here we consider possible roles for a condensate composed of +TIP proteins.

### Mechanism of MT dynamic instability and its regulation by MT binding proteins

To consider possible functions for +TIP condensates and how they might regulate MT dynamic instability (DI), it is first necessary to consider the mechanism of DI. MT growth and shrinkage result from the incorporation and detachment of tubulin dimers at protofilament ends exposed on MT tips. The details of the mechanism of DI are still being elucidated, but models center around the observation that tubulin subunits bind GTP but hydrolyze it soon after hydrolysis; this and other work have led to the idea that MTs can grow as long as they maintain a cap of GTP tubulin but switch to depolymerization (i.e., undergo “catastrophe”) if they lose this cap through stochastic GTP hydrolysis or other mechanisms (reviewed by [2]).

Additional detail in the process is provided by recent electron microscopy indicating that growing MTs have extended protofilaments that are laterally unbound near the tip [74]. This observation suggests that protofilaments need to “zip up” to be fully incorporated into the MT, and thus that the tip might be both fragile and very dynamic. Though surprising to some, the existence of laterally unbound protofilaments was predicted by a dimer scale computational model [75], and it is consistent with biophysical experiments that indicate that subunits exchange rapidly at the tip [76].

An important and puzzling observation is that MTs *in vivo* grow ~5x faster than those *in vitro*, even when tubulin concentrations have been matched [77]. Remarkably, mixtures of +TIPs (specifically EB1 and MAP215) can reconstitute *in vivo* rates of polymerization *in vitro* [7]. Two explanations have been offered for how the proteins are able to enhance the growth rates so much. One possibility is that MAP215 and its relatives enhance the addition of new tubulin subunits to the tip, e.g., by binding and “delivering” tubulin subunits [78]. The other possibility is that the proteins increase the rate of growth by reducing the fraction of tubulin subunits that detach after binding [4,79].

Though less intuitive, this second mechanism is attractive as an explanation for the dramatic effect of +TIPs, especially when combined with evidence that MT tips have laterally unbound protofilaments [74]. Briefly, the observation mentioned above that incoming tubulin subunits “exchange rapidly at the tip” means that most incoming subunits detach instead of becoming incorporated into the lattice [76]. A protein that could prevent detachment could dramatically increase the rate of polymerization [79]. Laterally unbound protofilaments as discussed above might be expected to break off in chunks. Thus, one straightforward way for +TIPs to enhance the net growth rate so dramatically would be to prevent detachment by zipping up the bonds between protofilaments [4].

### Hypothesis: The +TIP network forms a condensate that coats the MT tip, creating a superstructure that acts as a polymerization chaperone

**Fig 10:**
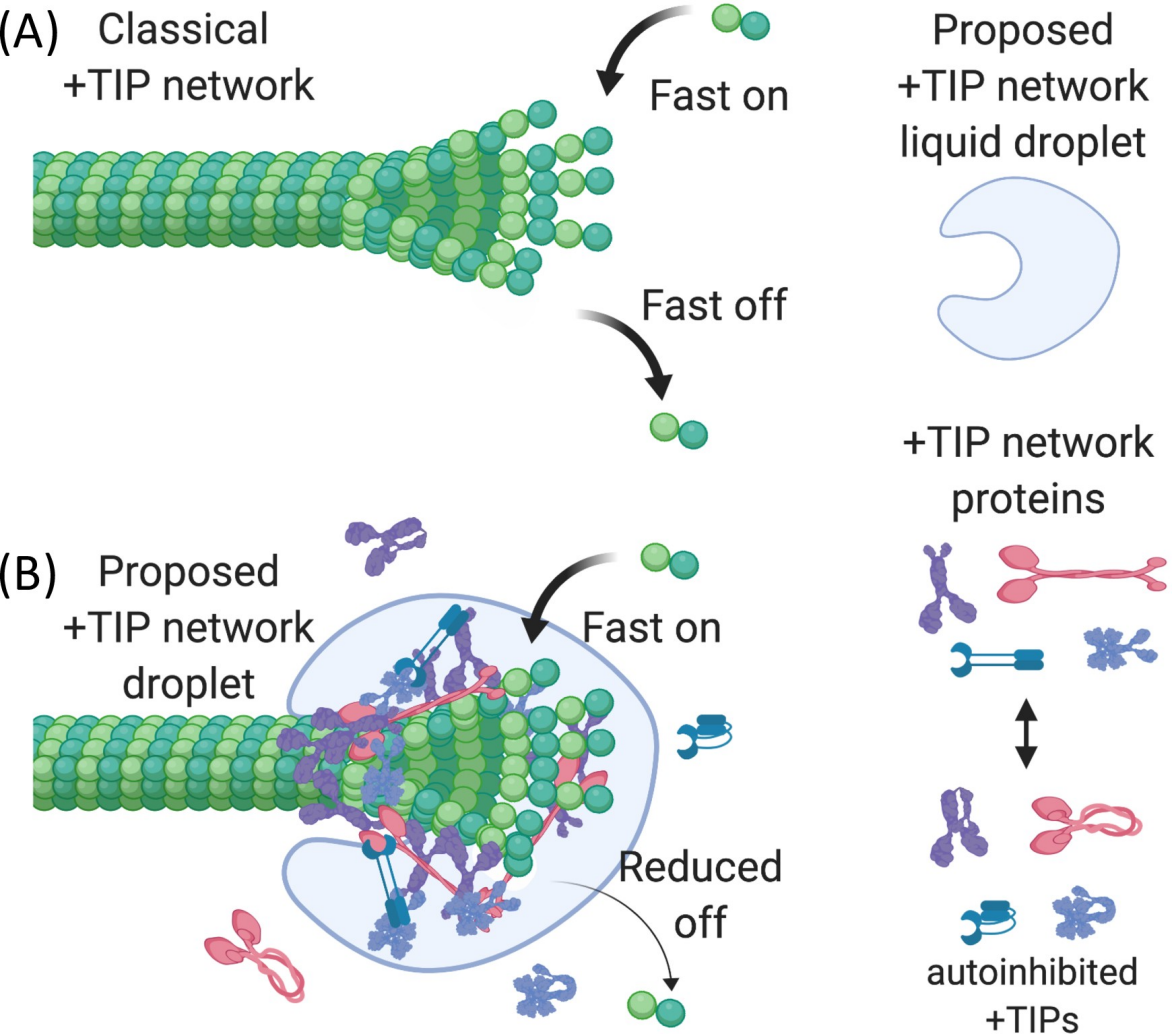
Proposed polymerization chaperone model. (A) In absence of the +TIP network, tubulin subunits arrive and leave quickly because most newly attached subunits are initially in the laterally unbonded regions of the tip protofilaments. The result is that MTs grow relatively slowly because most subunits detach before being incorporated into the laterally bound region of the MT. (B) In the presence of the proposed +TIP network liquid droplet, the web of interactions between the +TIPs creates a dynamic stocking-like structure that tracks the tip by diffusing on the potential energy gradient created by the preferential affinity of some tip components (most notably EB1) for tip-specific tubulin conformations. As the droplet moves, it exerts a force on the tip that promotes the zipping up of lateral bonds between protofilaments. This effect in turn increases the likelihood that newly arrived subunits are incorporated into the lattice and enables the MT to grow faster, as seen *in vivo* or with mixtures of +TIPs *in vitro* [4]. This figure was created with BioRender [5].

As mentioned above, our lab has previously proposed that the web of +TIP interactions may have a physical purpose, creating a superstructure that promotes MT polymerization by stabilizing the weak lateral interactions between protofilaments, thus increasing the fraction of incoming subunits that are incorporated into the lattice [4]. Here we extend this hypothesis by proposing that the +TIP network superstructure referred to in this original hypothesis is a biomolecular condensate. More specifically, we suggest that the endogenous +TIP network can form a stocking-like structure that acts as a polymerization chaperone by encircling the MT tip in a supportive web, increasing the rate of lateral bond formation between protofilaments, which in turn decreases the rate of subunit detachment and thus increases the overall rate of MT growth (Fig 10).

As indicated in Fig 10, such a structure could potentially track the growing end by diffusing on the “wave” formed by the GTP (or GDP-Pi) cap, e.g., as has been proposed for proteins such as MAP215 [80]. Alternatively, one could also imagine +TIP droplet superstructure having utility if placed in a specific static position on a MT, such as has been suggested for Tau condensates [36].

Whether +TIP comets necessarily correspond to condensates is not specified in our hypothesis. Indeed, one piece of evidence against the idea that all +TIPs comets involve condensates is that researchers performing FRAP of MT comets using diffraction-limited spots measured a t_1/2_ of ~0.2 sec [81]. This number is ~40 times faster than the t_1/2_ we observed for the CLIP-170 induced condensates (~8.5 sec). Regardless, even if the +TIP network on the average MT tip is not a condensate, one might expect that condensate formation could be triggered locally from +TIP-localized proteins in response to appropriate signals. One locale where the possibility of +TIP condensate formation seems particularly intriguing is at the kinetochore-MT attachment site, given that a number of +TIP proteins localize to kinetochores and that condensate formation at the kinetochore has already been suggested [82]. Indeed, the evidence cited above that condensates can generate mechanical force [66] is particularly striking when considered in the context of evidence that pulling forces on kinetochore components can promote MT growth [83,84].

In considering the potential function and regulation of +TIP condensate formation, it is notable that the CLIP-170-induced patches are frequently found away from MT plus ends (Figs 2 and 5, and S4 Fig in S5 File), and that they persist in the presence of the MT-depolymerizing drug nocodazole (Fig 5). Moreover, the observation that patches form in cells exposed to nocodazole after only eight hours of transfection (Fig 5) raises the possibility that patches may be able to form in the absence of MTs. These observations could be used to argue that the patches are independent of MTs, initially providing evidence against the polymerization chaperone model for condensate function.

However, it is important to remember that condensates generally have a critical concentration for assembly, meaning that when constituents are present at concentrations above this value, condensates form spontaneously [85]. Significantly, the value of this critical concentration could potentially be influenced by factors including the presence of a platform for assembly and/or post-translational modifications that influence the interactions between subunits of the condensate [85]. Thus, we explain the observation that patches exist apart from MT tips and persist (and perhaps even form) in nocodazole by speculating that under normal physiological conditions, the MT tip or some other structure (e.g, the kinetochore) acts as a platform to locally nucleate +TIP condensate formation. However, under CLIP-170 overexpression conditions, the critical concentration for spontaneous assembly is surpassed, allowing condensates to exist independently of MTs. How potential platforms (including MT tips) and/or regulatory mechanisms influence +TIP condensate formation is an interesting topic for future work.

## Conclusion

In summary, we have shown that “patches” induced by CLIP-170 overexpression are biomolecular condensates. The overexpression approach, although artificial, may still give us insight into the mechanism of the physiological +TIP network. Initial support for the idea that condensate formation is functionally significant is provided by our bioinformatics studies, which have shown that CLIP-170 and other members of the +TIP network have conserved IDRs. This evidence leads us to propose that the endogenous +TIP network can under appropriate conditions form a dynamic stocking-like condensate that zips up the lateral bonds between protofilaments to promote MT polymerization.

## Supporting information

S1 File Movie 1. Dynamic behavior of GFP-CLIP-170 in vivo when expressed at low levels. NIH3T3 cells were transiently transfected to express GFP-CLIP-170 for 24–27 hr, and the time-lapse images of 1 untransfected cell (number 0) and 4 transfected cells (numbers 1–4) were recorded by widefield microscopy. The numbers 1–4 correspond to the transfection level as assessed by the fluorescence intensity of the comets, with 1 and 4 indicating the lowest and highest levels of transfection respectively.
(AVI)

S2 File Movie 2. Micro-condensates appear in cells expressing medium-low levels of transfected GFPCLIP-170. Time-lapse images of a NIH3T3 cell expressing a medium-low level of GFP-CLIP-170 were recorded by widefield microscopy after 24–27 hr of transient transfection. Arrows indicate examples of apparent micro-condensates. However, it is difficult to distinguish comets from condensates.
(AVI)

S3 File Movie 3. Dynamic behaviors of GFP-CLIP-170 patches in cells (this movie corresponds to Fig 3). NIH3T3 cells were transiently transfected with GFP-CLIP-170, and the behavior of GFP-CLIP-170 in cells was recorded by confocal microscopy after 24–27 hr of transfection. Red box: An example of an apparent elastic deformation of a patch, followed by fission. Arrows indicate examples of patch fusion (magenta) and a photobleached site (green).
(AVI)

S4 File Movie 4. Dynamic behaviors of CLIP-170 patches in cells. NIH3T3 cells were transiently transfected with GFP-CLIP-170 for 24–27 of transfection. Time-lapse images of a cell expressing small CLIP-170 patches were recorded by widefield microscopy. The top arrow shows an example of comets going through and deforming a CLIP-170 patch. The bottom arrow shows an example of apparent micro-condensates.
(AVI)

S5 File Supplementary information. This file contains S1 Table and S1-S11 Figs, as well as the relevant references.
(PDF)

S6 File Supporting data for Fig 6 and S6 Fig in S5 File. This.zip file contains the minimal data sets used to reach the numerical conclusions drawn in Fig 6 and S6 Fig in S5 File.
(ZIP)
